# Spatial Analyses of Benthic Habitats to Define Coral Reef Ecosystem Regions and Potential Biogeographic Boundaries along a Latitudinal Gradient

**DOI:** 10.1371/journal.pone.0030466

**Published:** 2012-01-19

**Authors:** Brian K. Walker

**Affiliations:** Nova Southeastern University, National Coral Reef Institute, Dania Beach, Florida, United States of America; Université du Québec à Rimouski, Canada

## Abstract

Marine organism diversity typically attenuates latitudinally from tropical to colder climate regimes. Since the distribution of many marine species relates to certain habitats and depth regimes, mapping data provide valuable information in the absence of detailed ecological data that can be used to identify and spatially quantify smaller scale (10 s km) coral reef ecosystem regions and potential physical biogeographic barriers. This study focused on the southeast Florida coast due to a recognized, but understudied, tropical to subtropical biogeographic gradient. GIS spatial analyses were conducted on recent, accurate, shallow-water (0–30 m) benthic habitat maps to identify and quantify specific regions along the coast that were statistically distinct in the number and amount of major benthic habitat types. Habitat type and width were measured for 209 evenly-spaced cross-shelf transects. Evaluation of groupings from a cluster analysis at 75% similarity yielded five distinct regions. The number of benthic habitats and their area, width, distance from shore, distance from each other, and LIDAR depths were calculated in GIS and examined to determine regional statistical differences. The number of benthic habitats decreased with increasing latitude from 9 in the south to 4 in the north and many of the habitat metrics statistically differed between regions. Three potential biogeographic barriers were found at the Boca, Hillsboro, and Biscayne boundaries, where specific shallow-water habitats were absent further north; Middle Reef, Inner Reef, and oceanic seagrass beds respectively. The Bahamas Fault Zone boundary was also noted where changes in coastal morphologies occurred that could relate to subtle ecological changes. The analyses defined regions on a smaller scale more appropriate to regional management decisions, hence strengthening marine conservation planning with an objective, scientific foundation for decision making. They provide a framework for similar regional analyses elsewhere.

## Introduction

Latitudinal gradients have been identified as a biogeographic indicator for the large-scale distribution and diversity of marine organisms [1], [2], [3], [4], [5], [6], [7], [8]. Although the mechanisms are unknown [9], [10], the number of families, genera, and/or species specific to a tropical biogeographic zone generally decreases along a latitudinal gradient as it transitions into colder climate regimes [6], [7], [11], [12], [13], [14]. Biogeographic analyses are accomplished on a multitude of geographic and temporal scales depending on the system being studied; however, to provide a scientific basis for local marine conservation, it has been suggested that planning studies should focus on local or regional scales [15]. The absence of these data may 1) undermine marine spatial planning efforts due to a lack of understanding of the biotic relationships in the landscape, and 2) obfuscate relationships in scientific studies due to lack of the appropriate spatial information during sample site planning.

Remote sensing and management priorities have facilitated increased marine coastal ecosystem mapping in the last several years [16], [17], [18], [19], [20]. These maps allow regional inventories of marine habitats to be quantified as well as spatial analyses of the landscape to correlate with *in situ* data to indentify previously unattainable, larger-scale relationships [21], [22], [23], [24]. In the absence of detailed *in situ* data, spatial analyses of benthic habitat maps may fill an important role in identifying statistically distinct coral reef ecosystem regions based on habitat morphology.

Since the distribution of many marine species relates to certain habitats and depth regimes, mapping data provide valuable information that can be used to identify and spatially quantify smaller scale management regions and potential physical biogeographic barriers. High resolution Light Detection and Ranging (LIDAR) bathymetry and benthic habitat maps are useful tools for spatially analyzing coastal morphology extents and the spatial relationship of seafloor features [17], [22], [25]. LIDAR bathymetry gives detailed 3-dimensional perspectives of the seafloor and provides detailed depth information over broad areas [19], [26], [27]; while benthic habitat maps, in the form of geographic information system (GIS) vector data, facilitate the quantification of a feature's areal extent and its spatial relationship in the landscape [17], [22], [28].

The southeast Florida shallow-water (0–30 m) coastline is an ideal locale to apply such spatial analyses. Southeast Florida consists of several linear shore-parallel high-latitude coral reef communities extending north from the tropical Florida Keys. It has a linear shoreline with a recognized large-scale terrestrial biogeographic gradient transitioning from a tropical to a temperate Holdridge Life Zone [29] and several estuarine biogeographic zones have been identified [3].

Large-scale latitudinal biogeographic gradients on coral reefs have been reported worldwide [8], [14] and are evident for different ecological community aspects in several previous southeast Florida coral reef studies. A comprehensive literature review of the ecological functions of nearshore hardbottom habitats was recently conducted for the State of Florida [30]. Its focus was on habitats from 0 - 4 m depth, but it compiled deeper data as well. Although few data were available, obvious latitudinal changes in communities were found for a variety of species including a northward increase in macroalgae biomass and reduced ichthyofauna diversity [30]. The Southeast Florida Coral Reef Evaluation and Monitoring Project (SECREMP), presently the single-most comprehensive, consistent, regional coral reef research project in SE Florida [31], showed a northward attenuation of scleractinian coral species from Miami-Dade to Martin counties [31]. Within the limited number of SECREMP sites (17 throughout SE Florida), twenty-two coral species were present in Miami-Dade County, 21 in Broward, 18 in Palm Beach, and 5 in Martin. Although not representative of the entire scleractinian coral population in each county, the data suggest that the number of species lessens with increasing latitude in a methodologically consistent study. Furthermore, 17 scleractinian coral species were reported in St. Lucie Inlet Preserve State Park (Martin) [32] whereas at least 30 species exist in counties further south [31]. Increasing latitude corresponded to an increase in mean percent macroalgal cover and a higher abundance of *Diadema antillarum* as well.

Latitudinal and cross-shelf community differences have been reported in Broward County [33], [34]. Moyer et al. (2003) found an overall reduction in community diversity from south to north as well as reduced scleractinian coral and macroalgae cover and increased alcyonacian cover on the Middle and Outer reefs. They also found the Nearshore Ridge Complex (NRC) was statistically different from the Inner, Middle, and Outer reefs, having reduced benthic cover and topographic relief.

Latitudinal differences in scleractinian coral growth rates have been reported [34], [35]. Dodge (1987) found *Montastraea annularis* had higher growth rates in south Broward at 9 m depth than similar *M. annularis* colonies further north (north Broward), attributing this result to slightly warmer water and enhanced light availability in the south.

Latitudinal changes in the ichthyofaunal assemblages have been reported [30] where *Anisotremus surinamensis*, *Haemulon parra*, *Diplodus spp*., and *Labrisomus nuchipinnis* were found in significantly greater abundances in the North Palm Beach region [36] than further south in Broward [37]. Changes in the ichthyofaunal assemblages and decreased diversity are also evident within Palm Beach County [34].

Although marine faunal latitudinal differences have been recognized in SE FL, there are currently no synoptic regional survey data available to define separate regions within the larger area. Previous work along the southeast Florida coast has identified several distinct areas based on geomorphology [26], [38], yet an evaluation of the living coral reef communities and benthic habitats has not been performed. This study's objective was to apply a spatial analysis using recent, accurate benthic habitat maps to identify and quantify specific regions along the coast that are statistically distinct in the number and amount of major benthic habitat types. The analyses elucidate distinct regions based on the present-day coral reef community and seagrass morphologies that provides a scientific basis for local marine conservation spatial planning [15] and a framework for other similar regional analyses worldwide.

## Methods

### 2.1 Benthic Habitat Maps

**Figure 1:**
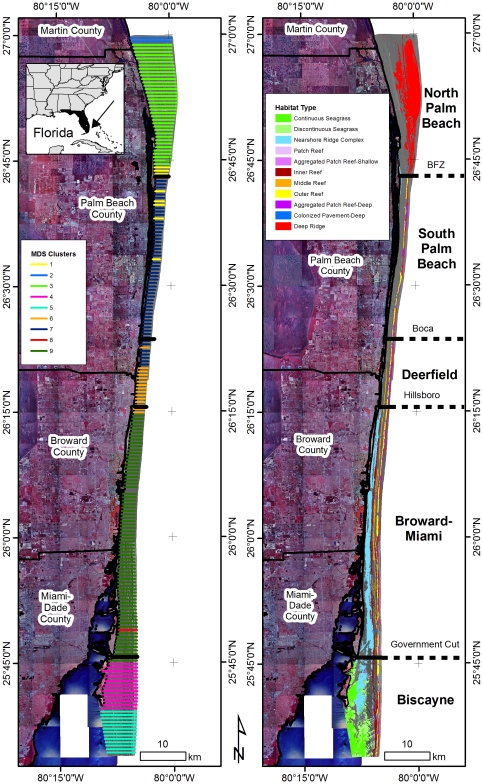
Overview maps showing the cross-shelf transects symbolized by the 75% similarity MDS clusters (left) and the five identified regions (right). BFZ = Bahamas Fault Zone.

Southeast Florida is comprised of four counties (listed from south to north): Miami-Dade, Broward, Palm Beach, and Martin (Figure 1). Existing benthic habitat maps for Miami-Dade, Broward, and Palm Beach counties were utilized for this work [17], [39], [40]. Habitat maps for Martin County were not complete at the time of the analysis.

Benthic habitat map creation for all counties involved a combined-technique approach incorporating LIDAR bathymetry, aerial photography, acoustic ground discrimination (AGD), video groundtruthing, limited subbottom profiling, and expert knowledge [17], [39], [40]. The maps were produced by visual interpretation of the high resolution LIDAR bathymetric data at a 1∶6000 scale with a 0.4 hectare minimum mapping unit and classifying the features based on their geomorphology and benthic fauna. *In situ* data, video camera groundtruthing, and acoustic ground discrimination were used to help substantiate the classification of the habitats. Accuracy assessment of the maps showed high levels of accuracy (>89%) which were comparable to that of using aerial photography in clear water [17], [40].

This study analyzed all mapped habitat types for coral reef and colonized hardbottom and seagrass. The following is a list of the habitat types and their definitions. The criteria for habitat classification were defined by their biologic communities, location, geomorphologic characteristics, and acoustic characteristics [17].

#### Coral Reef and Colonized Hardbottom

Substrates formed by the deposition of calcium carbonate by reef building corals and other organisms. Habitats within this category have some colonization by live coral.

**Nearshore Ridge Complex (NRC):** A combination of shallow colonized pavement and ridges found near shore in 3–5 m depth that are relatively flat, low-relief, solid carbonate rock. This habitat is dominated by a combination of scleractinian and octocorals, *Palythoa*, macroalgae, and sponges. Benthic coverage is highly variable but scleractinians are over 40% in some parts and several extensive monospecific aggregations *Acropora cervicornis* occur that are unique to the Florida Reef Tract [31].
**Inner Reef (IR):** A distinct, relatively continuous, shore-parallel reef that consists of a rich coral reef community which crests in approximately 8 m depth. The inner reef has an immature reef formation growing atop antecedent shallow colonized pavement. Acoustic and biological data indicates a distinct benthic community [33].
**Middle Reef (MR):** A distinct, relatively continuous, shore-parallel reef that consists of a rich coral reef community which crests in approximately 15 m depth. Acoustic and biological data indicate that it harbors a distinct benthic community from the NRC and IR [33].
**Outer Reef (OR):** A distinct, relatively continuous, shore-parallel reef that crests in approximately 16 m depth. It consists of a rich coral reef community living on relic reef morphology including a back reef, reef crest, and spur and groove. Acoustic and biological data indicate that it harbors a distinct benthic community [33], [41].
**Deep Ridge (DR):** Linear, often shore-parallel, low-relief features that mostly occur deeper than 25 m. It consists of hardbottom with sparse benthic communities in most parts likely due to variable and shifting rubble and sand cover. Acoustic data indicate a distinct benthic community [41].
**Patch Reef:** Coral or hardbottom formations that are isolated from other coral reef formations by sand, seagrass, or other habitats and that have no organized structural axis relative to the contours of the shore or shelf edge.

#### Seagrass

Habitat with 10 percent or more cover of *Thalassia testudinum* and/or *Syringodium filiforme*.

**Continuous Seagrass:** Seagrass community covering 90 percent or greater of the substrate. May include blowouts of less than 10 percent of the total area that are too small to be mapped independently (less than 4000 m^2^).
**Discontinuous Seagrass:** Seagrass community with breaks in coverage that are too diffuse, irregular, or result in isolated patches that are too small (less than 4000 m^2^) to be mapped as continuous seagrass.

#### Sand/Unconsolidated Sediments

Unconsolidated sediment with less than 10 percent cover of submerged vegetation.

### 2.2 LIDAR

Bathymetric data were used to determine average depth among the reef habitats and as the foundation for benthic habitat mapping. Three prior bathymetric surveys were conducted between 2002 and 2008 by Tenix LADS Corporation of Australia, using a LIDAR system with a sounding rate of 900 Hz (3.24 million soundings per hour), a position accuracy of 95% at 5-m circular error probable, a horizontal sounding density of 4 m×4 m, a swath width of 240 m, area coverage of 64 km^2^ h^−1^, and a depth range of 70 m, depending on water clarity. Vertical accuracy is depth dependant [42], however the reported error meets IHO SP44 (5th ed 2008) Order 1 standards [43], which, at 30 m depth (the maximum depth within the analysis), is less than ±0.6 m. The three surveys encompassed approximately 160 km linear north-south distance of southeast Florida from southern Martin County (27° N) to southern Miami-Dade County (25°35′N) from the shore eastward to depths of 40 m; approximately 600 km^2^ of marine seafloor. They were gridded in ArcGIS by the nearest neighbor algorithm and sun-shaded at a 45° angle and azimuth.

### 2.3 Spatial Analyses

Benthic habitat polygons were statistically tested for any spatial autocorrelation in ArcGIS using Moran's Index to determine any significant patterns in the underlying data significantly different from a random distribution.

Benthic habitat data were then statistically examined to determine where the number and size of seagrass, coral reef, and colonized hardbottom habitats significantly differ. Two-hundred and nine parallel, cross-shelf vector-line transects spaced approximately 750 m apart were created in GIS throughout the entire mapped region (Figure 1). An intersect was performed between the vector-line transects and the benthic habitat polygons, which broke the transect lines at each point where they intersected with a habitat polygon. The length of each resulting line segment was calculated to determine the linear cross-shelf distance of each habitat (width). A cluster analysis and corresponding non-metric multi-dimensional scaling (MDS) plot was then constructed using Bray-Curtis similarity indices (PRIMER v6) of the cross-shelf habitat width data (square-root transformed) to evaluate regions with distinct habitat composition. The groups of transects that occurred within the clusters with 75% similarity were then categorized in GIS and visually examined to evaluate the clusters for any spatial grouping consistency (Figure 1). Inspection of the benthic habitats where MDS clusters split helped identify the key locations in the habitat mapping data where the regional boundaries were defined. After defining the boundaries, all cross-shelf transects were categorized by the corresponding region. These categories were imported in Primer as factors and a one-way analysis of similarity (ANOSIM) was performed to statistically determine their similarity. The factors were also displayed on the MDS plot to see how the categorization related to the 75% MDS clusters.

Within each identified region, the planar areal extent of the Seagrass and Coral Reef and Colonized Hardbottom habitats polygons was calculated in ArcGIS 9.3. The mean distance among and between reef habitats and distance from shore was measured. Thirty parallel, evenly spaced, east-west cross-shelf vector line transects throughout each region were intersected with the benthic habitat polygons. The distances of the resulting line fragments were used to measure habitat width, distance from shore, and distance from Inner Reef. Mean benthic habitat depth was calculated by statistically summarizing all LIDAR depths within each habitat polygon for each region. Shapiro-Wilk W tests were performed to determine data normality. There was at least one case for each test (i.e. one habitat in one of the regions) where the W statistic was significant indicating a non-normal distribution, therefore all data were log transformed using the formula log_10_(x+1) to normalize the data. Transformed data met both normality and homogeneity assumptions for analysis of variance (ANOVA). One way ANOVA was used to separately test for significant differences in habitat width, habitat depth, distance from shore, and distance from Inner Reef between regions. Then a Student-Newman-Keuls (SNK) post-hoc test between means was performed. A *p* value<0.05 in both ANOVA and SNK were accepted as a significant difference. In all comparisons, *p* values were <0.001 unless otherwise noted.

## Results

Spatial autocorrelation tests on the benthic habitat polygon areas using Moran's Index did not show a pattern significantly different from random (Moran's I 0.006; z-score 0.204; p-value 0.838).

**Figure 2:**
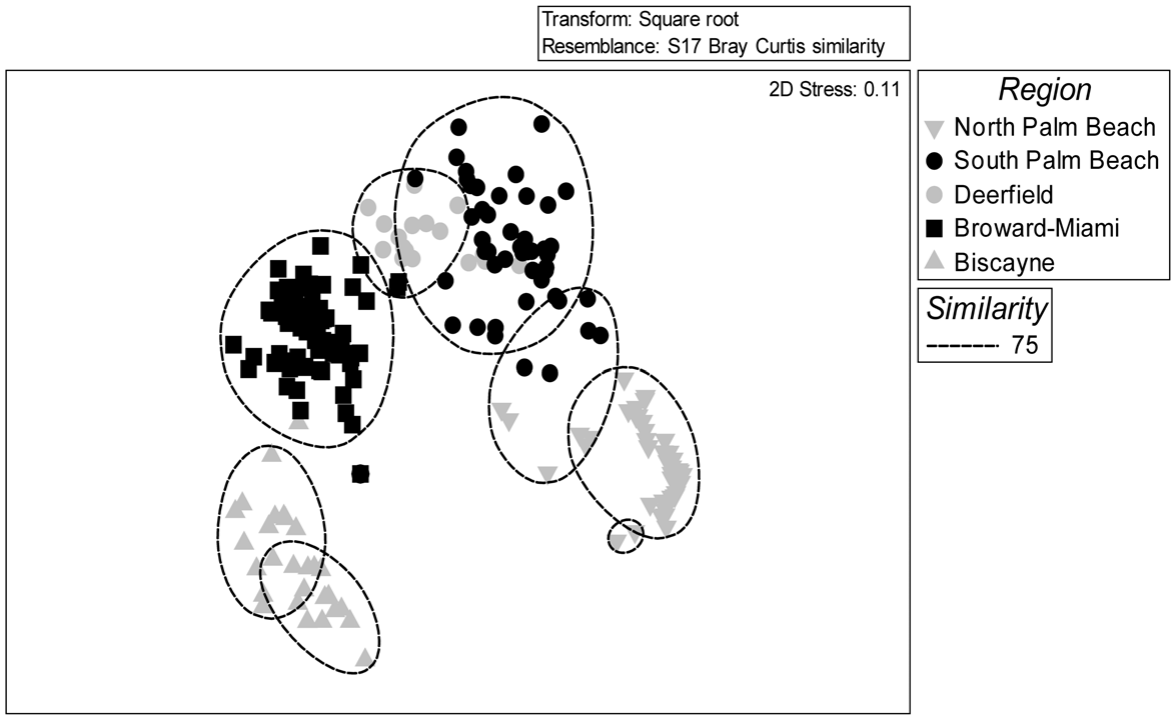
Multidimensional scaling (MDS) plot of Bray-Curtis similarity matrix of 209 regional cross-shelf transects displayed using the five final regional categories. The outlines represent 75% similarity from the cluster analysis.

Cluster analysis of the cross-shelf transects yielded nine clusters at the 75% similarity level and the two dimensional MDS plot showed relatively low stress (0.11) (Figure 2). Because the transects were placed at regular intervals without regard to the benthic habitats, they did not always adequately measure the local habitat morphology. Several of the transects crossed areas where there were gaps in one or more habitats, causing them not to cluster with other nearby transects that better-represented the local habitat morphology (e.g., Cluster 8 in Figure 1). Therefore, the cross-shelf transects categorized by the 75% MDS clusters were overlain in GIS onto the benthic habitat maps and inspected to identify the best location for the regional boundary. This yielded four boundaries that defined five regions (Figure 1). Two of these boundaries corresponded to present-day natural river inlets at Government Cut and Hillsboro Inlet; one corresponded to the Bahamas Fracture Zone, a previously identified fault line south of Lake Worth Inlet that marks the northern terminus of the Outer Reef [26], [38]; and one marked the northern terminus of the Middle Reef off Boca Raton, FL.

The analysis of similarity (ANOSIM) performed to statistically determine the similarity of the five final regions based on the cross-shelf transect data showed strong differences (R statistic>0.92) between categories in eight of the ten pairwise tests (Table 1). The regions that were most similar were the Deerfield and South Palm Beach regions (R statistic = 0.246). Inspection of the benthic habitat maps showed that the cross-shelf transects did not capture the presence of Middle Reef habitat in the northern part of the Deerfield region due to its fragmentation in that area, thus the northern transects in the Deerfield region were more similar to the transects in South Palm Beach.

**Table 1:** A summary of the analysis of similarity (ANOSIM) pairwise test between the five identified biogeographic regions.

*ANOSIM Pairwise Tests*	R	Significance
Groups	Statistic	Level %
Biscayne v. Broward-Miami	0.966	0.1
Biscayne v. Deerfield	0.999	0.1
Biscayne v. South Palm Beach	0.998	0.1
Biscayne v. North Palm Beach	1	0.1
Broward-Miami v. Deerfield	0.873	0.1
Broward-Miami v. South Palm Beach	0.966	0.1
Broward-Miami v. North Palm Beach	0.998	0.1
Deerfield v. South Palm Beach	0.246	0.1
Deerfield v. North Palm Beach	0.995	0.1
South Palm Beach v. North Palm Beach	0.924	0.1

The regions are less similar the closer the R statistic is to 1.

The number of major habitat types in the identified regions progressively increased from north to south from 9 in the Biscayne Region to 4 in the North Palm Beach Region (Table 2). The area and relative percentages of these major habitat types differed substantially between regions. They are presented here in order from north to south.

**Table 2:** A summary of the major habitat types in the six identified SE Florida latitudinal biogeographic transition regions.

	No. of Habitats	Total Area (rank)	Habitat Type	Area (km^2^)	% within Region	Mean Feature Width (m)	SD (m)	Mean Feature Depth (m)	SD (m)	Mean Distance from Shore(m)	SD (m)	Mean Distance from IR (m)	SD (m)
**North Palm Beach**	4	175.48 (1)	NRC	0.62	0.35%	132	103	3.4	2.0	42	63	-	-
			Patch Reef	0.12	0.07%	-	-	21.8	5.4	-	-	-	-
			Deep Ridge	74.23	42.30%	3,076	1,521	26.0	4.1	2,563	1,125	-	-
			Sand	100.51	57.28%	-	-	-	-	-	-	-	-
**South Palm Beach**	5	60.05 (4)	NRC	0.58	0.97%	125	87	4.0	1.5	165	87	-	-
			Patch Reef	0.04	0.07%	-	-	18.0	2.7	-	-	-	-
			Outer Reef	4.52	7.53%	179	78	18.1	3.2	1,336	171	-	-
			Deep Ridge	3.22	5.36%	154	95	28.2	4.2	1,600	205	-	-
			Sand	51.69	86.08%	-	-	-	-	-	-	-	-
**Deerfield**	6	25.27 (5)	NRC	0.37	1.46%	72	34	4.4	1.5	203	85	-	-
			Patch Reef	0.00	0.01%	-	-	25.6	5.4	-	-	-	-
			Middle Reef	1.74	6.88%	150	63	14.1	2.9	692	140	-	-
			Outer Reef	2.31	9.14%	164	71	19.0	2.6	1,378	137	-	-
			Deep Ridge	0.78	3.07%	155	73	33.8	3.2	1,658	88	-	-
			Sand	20.07	79.44%	-	-	-	-	-	-	-	-
**Broward-Miami**	7	167.53 (2)	NRC	49.31	29.43%	887	362	6.9	1.6	340	297	-	-
			Patch Reef	0.05	0.03%	-	-	12.3	4.3	-	-	-	-
			Inner Reef	12.18	7.27%	265	111	9.7	1.7	1535	572	-	-
			Middle Reef	9.21	5.50%	224	133	15.4	2.3	2111	765	277	138
			Outer Reef	9.61	5.74%	234	91	18.4	3.3	2594	605	912	154
			Deep Ridge	4.04	2.41%	106	63	29.9	3.5	2949	440	-	-
			Sand	83.13	49.62%	-	-	-	-	-	-	-	-
**Biscayne**	9	144.72 (3)	Cont. Seagrass	26.59	18.38%	-	-	4.6	1.6	-	-	-	-
			Discont. Seagrass	26.37	18.22%	-	-	-	-	-	-	-	-
			NRC	16.97	11.73%	1,253	724	6.4	1.1	1,607	493	1,943	640
			Patch Reef	0.31	0.22%	-	-	7.6	2.2	-	-	-	-
			Inner Reef	6.78	4.69%	368	192	8.2	2.2	5,089	616	-	-
			Middle Reef	0.29	0.20%	69	25	16.9	2.9	-	-	-	-
			Outer Reef	3.14	2.17%	220	153	16.3	4.5	5,899	787	386	304
			Deep Ridge	1.91	1.32%	106	65	31.5	4.8	6,337	803	-	-
			Sand	62.36	43.09%	-	-	-	-	-	-	-	

No. is the number of habitats in regions; Total Area (Rank) is the total planar area measured in GIS and its rank among all habitats from highest (1) to lowest (6).

### 3.1 North Palm Beach

**Figure 3:**
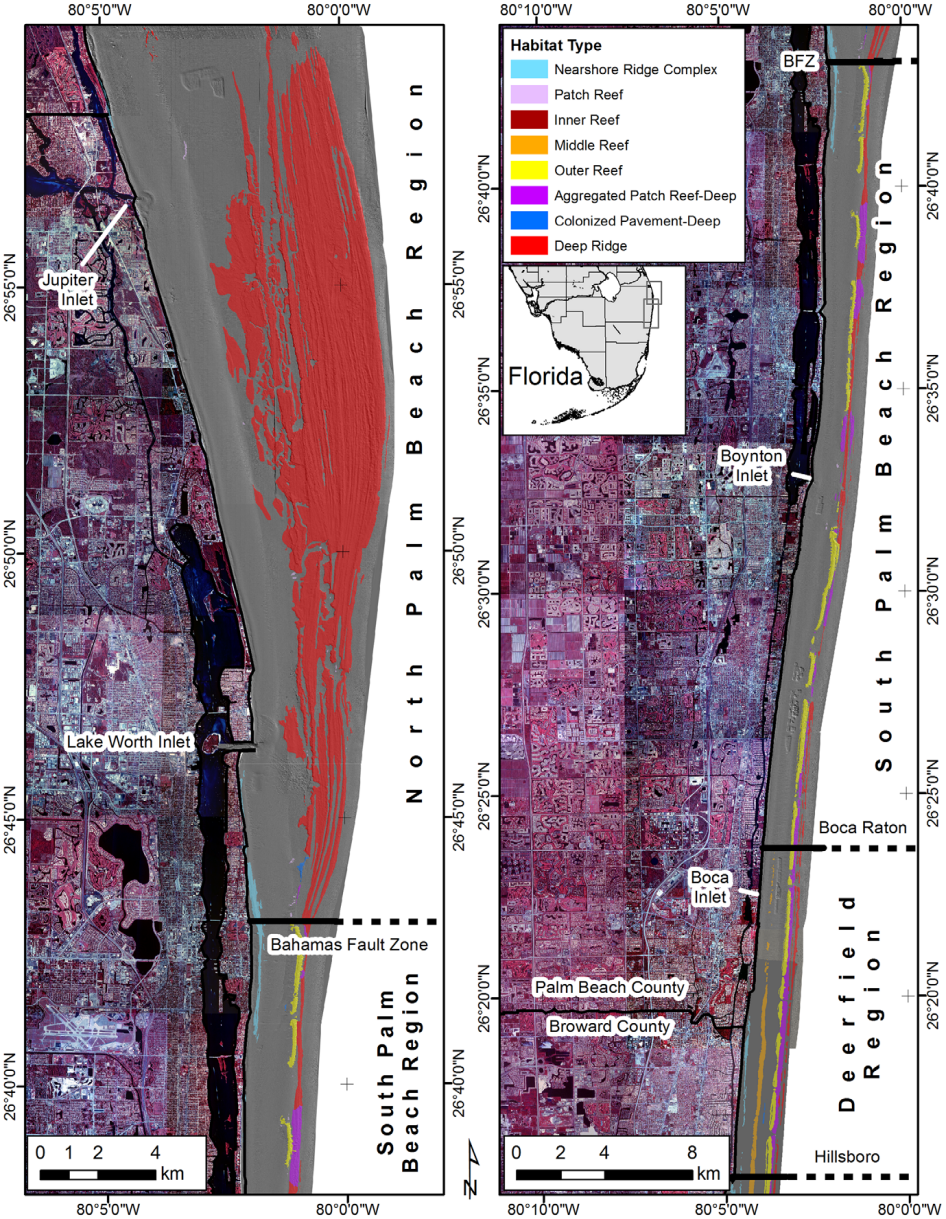
Coral reef habitats of the North Palm Beach, South Palm Beach, and Deerfield regions overlain on the hillshaded Lidar bathymetry (grey). Habitats are partially transparent to show feature relief. Horizontal black lines are the region boundaries. Map panels are not the same scale.

The North Palm Beach region spans approximately 32 km of coastline from the northern extent of the mapped area (27°N) south to the Bahamas Fault Zone (26°43′4.62″N) (Figure 3). This corresponded to Reach I in Finkl and Andrews (2008). The transition at the southern boundary of the North Palm Beach region marks the northern terminus of the Linear Reef-Outer, which is located just south of Palm Beach harbor [26], [38], [39]. This is also the point where the Florida current extends further from shore [3] and a widening of the coastal shelf is apparent [26]. Its lack of coral reef topography was conspicuous. The present-day coral communities in this region appear to be growing on cemented paleoshorelines [44] and not antecedent coral reefs.

**Figure 4:**
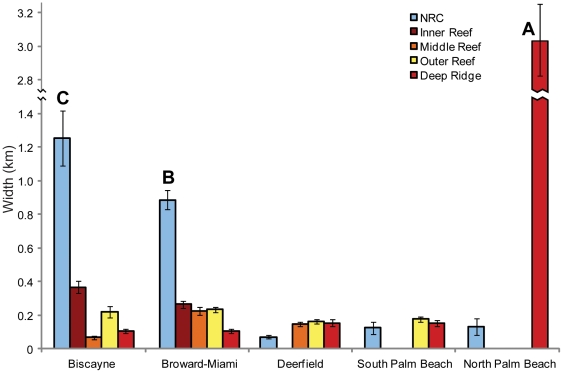
Mean habitat width by region. Vertical lines represent one standard error of the mean. Letters indicate significant differences detected by Student Newman Keuls post hoc test (p<0.001). Bars without letters are not significant from each other.

**Figure 5:**
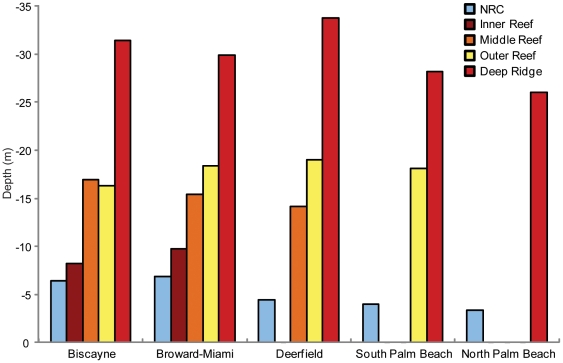
Mean habitat depth by region. One standard error of the mean was too small to be graphically depicted. All habitat depths were significantly different between regions (p<0.001). Bars without letters are significant from each other.

North Palm Beach ranked first among regions in size with a mapped area of 175.48 km^2^. It contained four major habitat types, Nearshore Ridge Complex (NRC), Patch Reef, Deep Ridge, and Sand, yet it was dominated by the latter two. North Palm Beach contained the most Sand (74.23 km^2^) and Deep Ridge (100.51 km^2^) habitats of the 6 regions and these two habitats comprised 99.58% of the area. NRC in this region was small (0.62 km^2^) and was limited to one place along the coast near the southern transition point. The Deep Ridge was significantly widest (3076 m±1521) (Figure 4) and shallowest (26.0 m±4.1) (Figure 5) in this region and the NRC was one of the shallowest (3.4 m±2.0) and thinnest (132 m±103). The Inner, Middle, and Outer Reefs and Seagrass were absent.

### 3.2 South Palm Beach Region

The South Palm Beach region (Figure 3) spans approximately 36 km of coastline from the Bahamas Fault Zone south to Boca Raton (26°23′40.78″N) similar to Reach II in Finkl and Andrews (2008); however, this region stopped 6.5 km north of Boca Inlet instead of at Boca Inlet. The boundary at Boca Raton marks the northern terminus of the Middle Reef.

**Figure 6:**
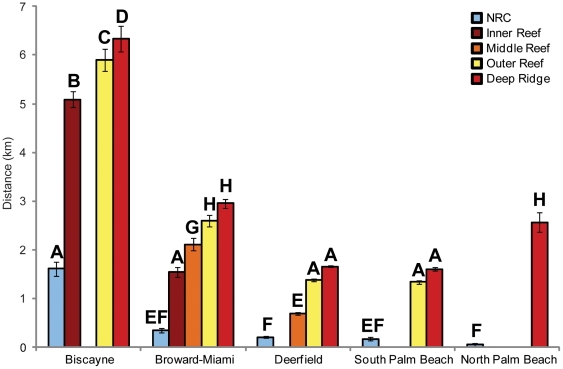
Mean habitat distance from shore by region. Vertical lines represent one standard error of the mean. Letters indicate significant differences detected by Student Newman Keuls post hoc test (p<0.05). Bars with the same letters are not significant from each other.

South Palm Beach ranked fourth in size with a mapped area of 60.05 km^2^ and contained five major habitat types. The Outer Reef was a conspicuous feature in the region ranking second among the regions in planar area (4.52 km^2^) behind Broward-Miami. South Palm Beach had one of the thinnest shelf widths evinced by the Outer Reef (1336 m±171) and Deep Ridge (1600 m±205) being significantly closer to shore than all regions except Deerfield (Figure 6). The mean distance from shore of the Outer Reef in South Palm Beach was not statistically distinct from Broward-Miami's Inner Reef (1326 m±360) and Biscayne's NRC (1606 m±493). South Palm Beach contained the second highest percentage (5.36%; 3.22 km^2^) of Deep Ridge but very little NRC (0.97%; 0.58 km^2^) and Patch Reef (0.07%; 0.04 km^2^). Most notable in this region were the small amount of NRC and the absence of Middle Reef, Inner Reef, and Seagrass habitats.

### 3.3 Deerfield Region

The Deerfield Region spans approximately 15 km of coastline bounded by the Boca Raton boundary and Hillsboro Inlet (26°15′32.73″N) (Figure 3). This ranked as the smallest of the regions with a mapped area of 25.27 km^2^. Deerfield contained six major habitat types. The southern boundary marked the northern terminus of the Inner Reef which has been previously identified in a geologic context [26], [38].

Although this region had the second highest percentage of Sand (79.44%; 20.07 km^2^), the Middle Reef and Outer Reef were both conspicuous habitats comprising the highest percentage of all the regions; 6.88% (1.74 km^2^) and 9.14% (2.31 km^2^) respectively. It contained 3.07% Deep Ridge (0.78 km^2^) and 1.46% NRC (0.37 km^2^). The Middle Reef was significantly closest to shore than in any other region (692 m±140) (Figure 6), but did not statistically differ in distance to shore from Broward-Miami NRC (340 m±297) (p = 0.09) and South Palm Beach NRC (165 m±87) (p = 0.053). The Inner Reef and Seagrass habitats were absent in this region.

### 3.4 Broward-Miami Region

**Figure 7:**
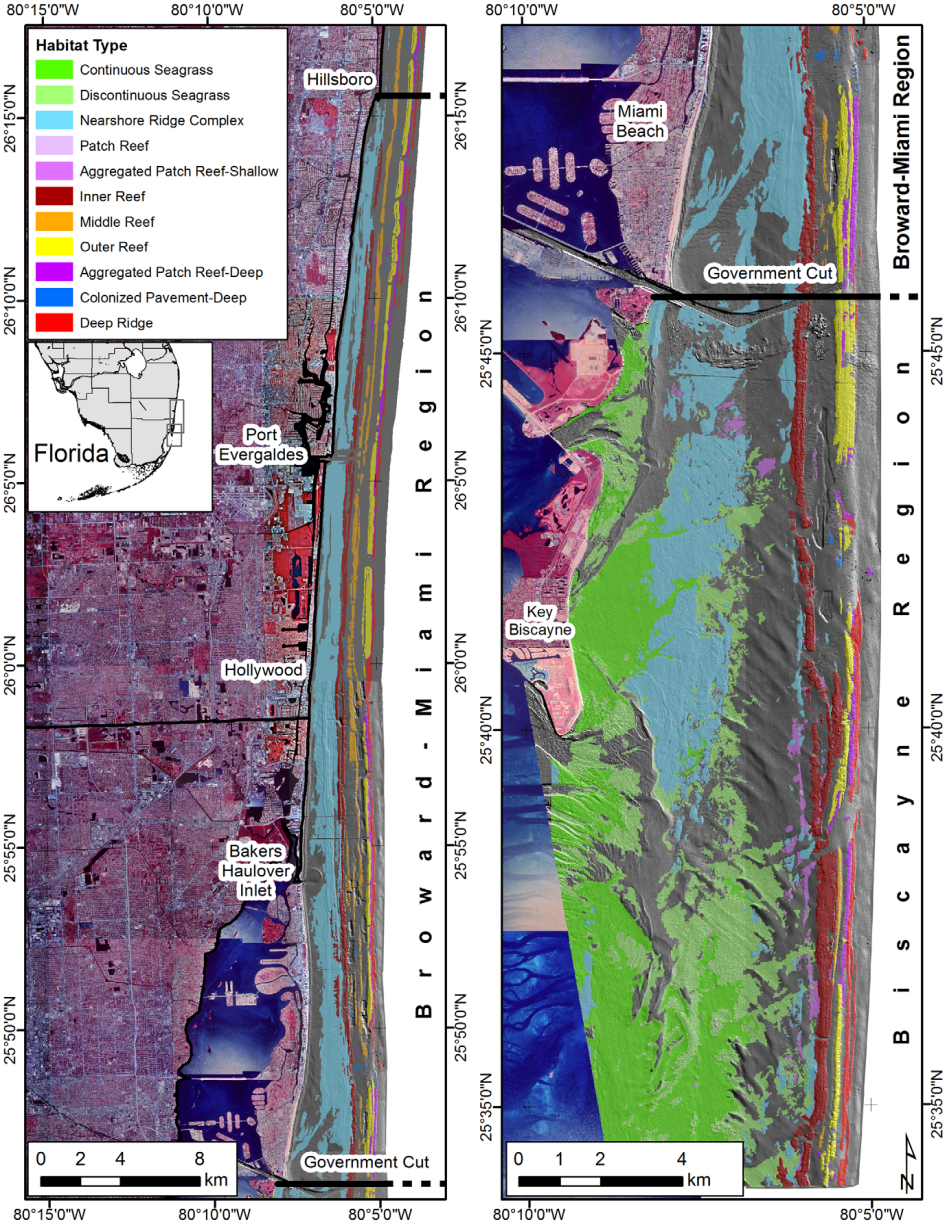
Coral reef habitats of the Broward-Miami and Biscayne regions overlain on the hillshaded LIDAR bathymetry (grey). Habitats are partially transparent to show feature relief. Horizontal black lines are the region boundaries. Map panels are not the same scale.

The Broward-Miami Region (Figure 7) spans approximately 48 km of coastline bounded by the Hillsboro inlet (north) and Government Cut (25°45′44.35″N), mostly corresponding with Reach III of Finkl and Andrews (2008). It ranked second in size with an area of 134.67 km^2^ and contained seven major habitat types. The shelf here was wider than in South Palm Beach and Deerfield as evidenced by the significantly greater distance from shore of the Middle Reef (2111 m±765), Outer Reef (2594 m±605), and the Deep Ridge (2949 m±440). Although Sand was dominant (49.62%; 83.13 km^2^), Broward-Miami contained high percentages of NRC (29.43%; 49.31 km^2^), Inner Reef (7.27%; 12.18 km^2^), Middle Reef (5.50%; 9.21 km^2^), Outer Reef (5.74%; 9.61 km^2^), and Deep Ridge (2.41%; 4.04 km^2^). Broward-Miami NRC was significantly wider than NRC in Deerfield, South Palm Beach, and North Palm Beach (Figure 4). Seagrass habitats were absent.

Interestingly, the mean distance between the Inner and Outer reefs significantly decreased from 912 m (±154) in Broward-Miami to 386 m (±304) in Biscayne. Further inspection of the habitat maps showed that the two features began converging north of Government Cut at the same latitude (25°49′55.31″N) where examination of pre-developed shoreline maps showed a previous natural river inlet named Boca Ratones mapped by DeBrahm in 1770 that closed pre-1887 [45], [46], [47], [48]. The reefs actually converge in the Biscayne region further south off Key Biscayne where the Inner Reef appears to be growing atop the Outer Reef. Although not of management or biogeographic significance, this convergence of the Inner and Outer reefs has not yet been reported and could be of importance to future geologic studies.

### 3.5 Biscayne Region

The Biscayne Region (Figure 4) spans approximately 22 km of coastline bounded by Government Cut (north) and the end of the mapped area (south) (25°35′N), corresponding to Reach IV in Finkl and Andrews (2008). This region ranked third in size with an area of 144.72 km^2^ and contained nine major habitat types. The boundary at Government Cut marked the northern extent of known seagrass beds on the ocean side of the coast. Although Sand was dominant (43.09%; 62.36 km^2^), Continuous (18.38%; 26.59 km^2^) and Discontinuous Seagrass (18.22%; 26.37 km^2^) combined comprised 36.6% of the mapped area.

Biscayne contained a large amount of NRC (11.73%; 16.97 km^2^) and Inner Reef (4.69%; 6.78 km^2^) but their proportions were lower than Broward-Miami. Moderate amounts of Outer Reef (2.17%; 3.14 km^2^) and Deep Ridge (1.32%; 1.91 km^2^) were present and although small, the Patch Reef habitat area (0.22%; 0.31 km^2^) ranked first between all regions. The Middle Reef was barely evident (0.2%; 0.29 km^2^).

The Biscayne shelf was wide and the reef habitats were significantly farther from shore than their respective counterparts in all other regions (Figure 6). The mean distance from shore (Key Biscayne/Atlantic Ridge) of the Outer Reef (5899 m±787) was 56% farther than Broward-Miami, 77% farther than Deerfield and South Palm Beach. The NRC was significantly widest in Biscayne than all other regions (Figure 4).

Statistical differences were detected in all habitat depths between regions. Some notable differences were that the NRC was deepest in Broward-Miami and Biscayne than northern regions (Figure 5). Mean Inner Reef (8.2±2.2 m) and Outer Reef depths (16.3±4.5 m) were shallowest in Biscayne. Also, Patch Reefs were shallower in Biscayne (7.6±2.2 m) than any other region and Broward-Miami patch reefs (12.3±4.3 m) were deeper than more northern regions (Table 2).

## Discussion

### 4.1 Region boundaries as potential biogeographic barriers

In the Western Atlantic, large-scale biogeographic boundaries have been determined by zoogeographic provinces and climatic zones with temperature being one of the most important factors [49]. However, many other causative factors may be limiting organismal distributions, including physical and/or spatial barriers [3], [7], [13], [50], [51], [52]. In benthic marine systems, changes in coastal morphologies may cause physical barriers beyond which suitable conditions may not exist for habitat-specific organisms. Previously, cold water temperature limitations have been attributed to the “rapid diminution of generic diversity northwards along the east coast of Florida” [53]. The analyses herein show that a latitudinal benthic habitat zonation also exists along southeast Florida, where the size and number of distinct benthic habitats lessened northward. Combined with the coincident latitudinal changes in temperature and current regimes, the loss of specific benthic habitats with increasing latitude is likely a spatial barrier for its associated fauna.

Regional boundaries that appeared to be major spatial barriers were located at the Biscayne, Hillsboro, and Boca transitions, where the northern extent of distinct habitats was detected, and a potential barrier was noted at the Bahamas Fault Zone where changes in habitat morphologies occurred that may relate to subtler regional ecological differences.

The Biscayne region is the northernmost area where large *Thalassia* and *Syringodium* seagrass meadows exist seaward of the coastline. These meadows are highly productive and serve many ecological functions including the production of significant carbon material, sediment stabilization, water baffling, and providing habitat for many species of fish, crustaceans, and mollusks; especially juveniles [54], [55], [56], [57]. *Thalassia* leaves provide suitable substratum for epiphyte attachment and growth of a wide variety of algae, invertebrates, and microscopic organisms [58]. And similar to dune vegetation, these meadows help maintain shorelines with deep root and rhizome structures that reduces erosion, particularly in high energy areas [54]. The northern Biscayne region boundary at Government Cut is the northern biogeographic limit for shallow seaward tropical Atlantic seagrass meadows and their exclusively associated species and functions. *Thalassia* and *Syringodium* seagrasses do occur further north along the Atlantic coast, but are almost exclusive to the estuaries [59]. Differences in the ecological roles between tropical and temperate seagrass meadows are not well documented but comparisons have shown significant differences in species and generic diversity, community composition, and temporal variations [60]. The inshore seagrasses further north are limited to very shallow, protected, lower temperature environments and likely have different associated communities.

The Hillsboro Inlet marked a major transition in habitat morphology where the Inner Reef and extensive expanses of Nearshore Ridge Complex terminated and thus defined the boundary between the Broward-Miami and Deerfield regions. It remains unknown whether this is the end of these features or if they extend northward buried under the coastline [38], but they cease to function as coral reef habitat. The absence of available shallow hardbottom habitat affects benthic organism settlement distributions and results in “a significant reduction of high biomass of invertebrates (sponges, corals, crabs, shrimp, worms, gastropods, bivalves) that could significantly change the nearshore food web” [30]. The NRC has statistically different benthic and fish populations than the Middle and Outer reefs [24], [33] and is a recognized important habitat for juvenile fishes where disproportionately high abundances have been found [34], [36], [37], [61]. The loss of NRC affects settlement patterns of reef fish and thus changes the reef fish population structure, which may contribute to the observed latitudinal differences in fish communities by CSA International Inc. (2009). Therefore, the shallow-water reef communities and their associated ecological contributions recognized further south (Broward-Miami and Biscayne) are greatly reduced in the Deerfield region due to the lack of significant NRC and Inner Reef.

The Boca transition defined the boundary between the Deerfield and South Palm Beach regions because this location marked where all significant amounts (>1 acre) of NRC and Middle Reef end; 6.5 km north of Boca Inlet. Northward of this transition, there is a 36 km stretch of coast without significant hardbottoms outside of the intertidal zone that are shallower than 15 m depth. Thus the ecosystem functions associated with the NRC (that are presumably drastically reduced in the Deerfield region as discussed above) are absent in South Palm Beach, making it a biogeographic barrier for fauna specific to these habitats.

The Bahamas Fault Zone transition was distinct by the obvious change in habitat morphology between the South Palm Beach and North Palm Beach regions. Although not an obvious spatial barrier because coral reef habitat occurs in similar depths in both regions, feature morphology (and perhaps the composition) is quite different. Support for a spatial barrier here may be found in the ichthyofauna. An analysis of 2440 surveys showed, of the 400 total species seen, 43 species were seen exclusively in the North Palm Beach region and 56 exclusively in the South Palm Beach region [34]. Latitudinal and cross-shelf differences in fish species richness between these regions may exemplify differences in the benthic habitat communities between the north and south [34].

Coincident with coastal morphology and potential spatial barriers, there is an obvious change in water temperatures and currents from Biscayne to North Palm Beach [34]. The northward flowing warm waters of the Florida current that bathe the southern regions diverge from the coast near the Bahamas Fault Zone [3]. Monthly surface water temperatures vary latitudinally and temporally, but are consistently lower further north [31], [62]. For example, in 2008–2009, Miami Beach (southern Broward-Miami region) monthly-averaged surface water temperatures ranged from 21.7°C in Jan to 30°C in July, whereas Stuart Beach (north of the North Palm Beach region) temperatures ranged from 19.4°C in January to 27.2°C in September [63]. Deep upwelling colder water regularly occurs along the coast and appears more frequently in the north [30]. Frequent cold water pulses are evident on the reefs and mean daily temperatures differ nearly 4°C between Miami-Dade (24.5°C) and Martin (20.8°C) counties for the same period [31]. It is likely that drastic water temperature differences along this relatively short coastline is a large cause for latitudinal flora and fauna differences, however major morphologic changes in the seascape also contribute. The absence of shallow water hardbottom in the South Palm Beach region creates a spatial barrier for all shallow-water-habitat-associated species where NRC communities further north (in the North Palm Beach region) are noticeably different likely due to more frequent interaction with colder water.

### 4.2 Implications of spatial barriers on range expansion

Spatial barriers coincident with significant changes temperature and currents could have an influence on short-term range expansion of benthic species. Warmer temperatures have shifted the ranges of many species worldwide and are expected to continue over the next century [64], [65]. Previous studies have shown that coral reef poleward range shifts have occurred in warmer periods [66], [67] and may be occurring now [53], [68], however coastal morphology must support such shifts. As this study elucidates, in SE Florida there is little exposed structure for shallow (<15 m) coral communities to recruit to in a poleward range expansion.

Historically in southeast Florida, reefs initiated on beach ridges inundated by coastal flooding during the Holocene transgression [38], [44], [69], [70]. As sea level rose, it flooded the coastlines and submerged cemented beach dunes upon which corals subsequently grew. Presently, coastal development and the regular practice of beach renourishment impede the natural coastline erosion and flooding process, thus impeding new available substrate for colonization. The lack of significant Nearshore Ridge Complex present in the South Palm Beach region and increased sedimentation from beach nourishments will be a large hurdle for many shallow-water coral reef species to overcome in a poleward expansion along the southeast Atlantic coast.

For example, the threatened staghorn coral *Acropora cervicornis* has likely increased density and cover in southeast Florida over the past few decades, making it a dominant part of localized NRC coral communities [71], [72]. It mostly occurs in the shallow water hardbottom communities of the NRC and Inner Reefs with very few, small colonies found deeper than 15 m. This species has been proposed as a recent poleward-expansion candidate [53]. Due to the gap in shallow-water habitats further north, their proximity to shore, and their very shallow depths, the Boca transition will likely mark the northern extent of extensive shallow-water *A. cervicornis* communities until climate change has significantly altered the shallow water conditions in the North Palm Beach region to support this species.

### 4.3 Historical Perspective

Previous geologic analyses have shown that Outer Reef growth in southeast Florida terminated approximately 8 ka (Lighty et al., 1978; Banks et al., 2007). Hypotheses of the inability for historical reef accretion to keep up with rising sea-level include rate of sea-level-rise (Fairbanks, 1989; Blanchon and Shaw, 1995), the introduction of inimical waters from the flooding of coastlines (Lighty et al., 1978), and a Caribbean-wide reduction in reef building corals (Banks et al., 2007). Whatever the cause, most of the Outer Reef, which once resembled a classic cresting *Acropora palmata*-dominated Caribbean reef, now resides in much deeper water.

Analyses of present-day reef depths between regions revealed that the Outer Reef is significantly shallower in Biscayne than more northern regions (Figure 5). Present-day Outer Reef mean depths in the Biscayne region were over 2 m shallower. This outcome indicates that either historical erosion or reef growth varied between regions.

Increased erosion in the north is possible due to the location of the Florida Current. Historical Florida Current location and rates are unknown, but the distance between the Outer Reef and present Florida Current lessens northward along the coast as the shelf thins. Assuming the historical current was similar in location and strength, it is plausible that the northern Outer Reef had more interaction with the current over the past 8,000 years and thus eroded more.

Variable historical reef growth could also explain latitudinal differences in depth along the Outer Reef. This elicits two scenarios: 1) reef accretion terminated earlier in the north or 2) accretion occurred at a slower pace in the north. These growth scenarios suggest that climatic variability along the southeast Florida latitudinal gradient affected reef growth during the Holocene. Previous Outer Reef ages came from one site near the Hillsboro transition (Lighty et al., 1978), thus latitudinal variability of reef growth and termination ages along the northern Florida reef tract remain unknown.

The first historical growth scenario may be supported by present-day reef morphology. In the Biscayne region (where the Outer Reef is shallowest), the Inner and Outer Reefs appear to converge offshore of Key Biscayne (Figure 4). Here the Inner Reef grows immediately adjacent to and perhaps on top of the Outer Reef. This may be an area where reef accretion did not terminate 8,000 years ago. Geologic confirmation is needed on latitudinal differences in reef thicknesses and ages to determine how they relate to historical reef growth. If confirmed, it may be that historical reef growth did not simultaneously terminate along the northern extent of the Florida reef tract as previous research has indicated.

### 4.4 Marine Spatial Planning

Globally, the area of mapped coastal marine ecosystems is increasing. For example, in the last 10–15 years the United States (US) National Oceanic and Atmospheric Administration has mapped over 9,000 km^2^ of shallow-water coral reef benthic habitats within the US, its territories, and commonwealths spanning the Caribbean and Pacific oceans. The areas mapped thus far include Hawaii and the northwest Hawaiian Islands, American Samoa, Guam, Commonwealth of the Northern Mariana Islands, Palau, Palmyra, Puerto Rico, and the US Virgin Islands. The spatial analyses used herein could be applied to any large-scale mapping effort to statistically determine distinct coral reef ecosystem regions and potential biogeographic boundaries. The outcomes of which would strengthen scientific research by informing the appropriate spatial information during research sample site planning and allow for samples to be randomly stratified across regions and habitats based on local habitat morphology.

Furthermore, the analyses herein provide a scientific basis for local marine conservation spatial planning. According to Lourie and Vincent (2004) “…biogeography should be at the forefront of determining spatial priorities for proactive marine conservation planning. The spatial distribution and scale of biodiversity, the processes maintaining it, and the threats to it need to be understood so that appropriate conservation measures may be initiated.” The analyses herein defined regions at a scale appropriate to regional management decisions that relate to benthic habitat morphology and potentially to regional biogeography. This information will strengthen systematic marine conservation planning by furnishing necessary, relevant spatial distribution information that provides an objective, scientific foundation for decision making. As more regional biological data become available, the regions defined herein can be tested to better understand how the benthic fauna and ichthyofauna composition differ and how they are affected by differences in the major spatial relationships and sea floor morphologies.
